# Childhood interleukin-6, C-reactive protein and atopic disorders as risk factors for hypomanic symptoms in young adulthood: a longitudinal birth cohort study

**DOI:** 10.1017/S0033291716001574

**Published:** 2016-08-01

**Authors:** J. F. Hayes, G. M. Khandaker, J. Anderson, D. Mackay, S. Zammit, G. Lewis, D. J. Smith, D. P. J. Osborn

**Affiliations:** 1 Division of Psychiatry, UCL, London, UK; 2 Department of Psychiatry, University of Cambridge, Cambridge, UK; 3 Institute of Health and Wellbeing, University of Glasgow, Glasgow, UK; 4 Institute of Psychological Medicine and Clinical Neurosciences, Cardiff University School of Medicine, Cardiff, UK; 5 Centre for Academic Mental Health, School of Social & Community Medicine, University of Bristol, Bristol, UK

**Keywords:** Atopic disorders, cohort studies, hypomania, inflammation

## Abstract

**Background:**

There are no existing longitudinal studies of inflammatory markers and atopic disorders in childhood and risk of hypomanic symptoms in adulthood. This study examined if childhood: (1) serum interleukin-6 (IL-6) and C-reactive protein (CRP); and (2) asthma and/or eczema are associated with features of hypomania in young adulthood.

**Method:**

Participants in the Avon Longitudinal Study of Parents and Children, a prospective general population UK birth cohort, had non-fasting blood samples for IL-6 and CRP measurement at the age of 9 years (*n* = 4645), and parents answered a question about doctor-diagnosed atopic illness before the age of 10 years (*n* = 7809). These participants completed the Hypomania Checklist at age 22 years (*n* = 3361).

**Results:**

After adjusting for age, sex, ethnicity, socio-economic status, past psychological and behavioural problems, body mass index and maternal postnatal depression, participants in the top third of IL-6 values at 9 years, compared with the bottom third, had an increased risk of hypomanic symptoms by age 22 years [adjusted odds ratio 1.77, 95% confidence interval (CI) 1.10–2.85, *p* < 0.001]. Higher IL-6 levels in childhood were associated with adult hypomania features in a dose–response fashion. After further adjustment for depression at the age of 18 years this association remained (adjusted odds ratio 1.70, 95% CI 1.03–2.81, *p* = 0.038). There was no evidence of an association of hypomanic symptoms with CRP levels, asthma or eczema in childhood.

**Conclusions:**

Higher levels of systemic inflammatory marker IL-6 in childhood were associated with hypomanic symptoms in young adulthood, suggesting that inflammation may play a role in the pathophysiology of mania. Inflammatory pathways may be suitable targets for the prevention and intervention for bipolar disorder.

## Introduction

The role of inflammation in a variety of mental disorders has become a topic of renewed interest (Krishnadas & Cavanagh, 2012; Harrison, 2013; Kiecolt-Glaser *et al.* 2015). There is now considerable evidence that acute psychotic, manic and depressive episodes are associated with abnormalities in a range of inflammatory markers, including elevation of proinflammatory cytokines (Frommberger *et al.* 1997; Dickerson *et al.* 2007; Goldstein *et al.* 2011; Berk *et al.* 2013; Khandaker *et al.* 2015). There is also evidence that these disorders are longitudinally associated with previous inflammatory illnesses, for example maternal and childhood infections have been identified as risk factors for schizophrenia (Khandaker *et al.* 2012, 2013) and bipolar disorder (Parboosing *et al.* 2013). Individuals with psychotic and affective illnesses are also at increased risk of developing conditions with an inflammatory basis, such as diabetes and cardiovascular disease (Osborn *et al.* 2008; De Hert *et al.* 2009). However, there is a paucity of longitudinal research investigating whether alterations in inflammatory markers occur as a precursor to mental disorders. Data from the Avon Longitudinal Study of Parents and Children (ALSPAC), a UK general population birth cohort, recently showed that higher serum levels of interleukin-6 (IL-6) in childhood is associated with depression and psychosis in young adult life (Khandaker *et al.* 2014*a*).

ALSPAC data have also shown an association between childhood atopic disorders and psychotic symptoms in adolescents (Khandaker *et al.* 2014*b*). A longitudinal association between asthma and subsequent hospitalization for schizophrenia, and adult diagnoses of depression has been reported (Timonen *et al.* 2003; Pedersen *et al.* 2012; Sanna *et al.* 2014), and three longitudinal studies have reported increased rates of bipolar disorder in individuals with asthma (Liang & Chikritzhs, 2013; Chen *et al.* 2014; Lin *et al.* 2014). IL-6, C-reactive protein (CRP) and other inflammatory markers are associated with asthma and eczema (Takemura *et al*. 2006; Arif *et al*. 2007; Rincon & Irvin, 2012). However, to our knowledge there have been no previous studies showing temporal associations between inflammatory markers, atopic conditions and features of hypomania. Indeed it may be that this association is less likely than a longitudinal association with psychotic experiences as, whilst it has been found that chronic immune activation occurs in schizophrenia, previous research has suggested that bipolar disorder is an episode-related inflammatory syndrome, with normal cytokine function between affective episodes (Kunz *et al.* 2011; Stertz *et al.* 2013). Hypomanic symptoms in early adulthood are a risk factor for developing bipolar disorder (Zimmermann *et al.* 2009), but are also associated with increased future risk of non-affective Axis I disorders, personality disorders, mental health service use and psychotropic drug prescribing (Päären *et al.* 2014).

**Fig. 1.**
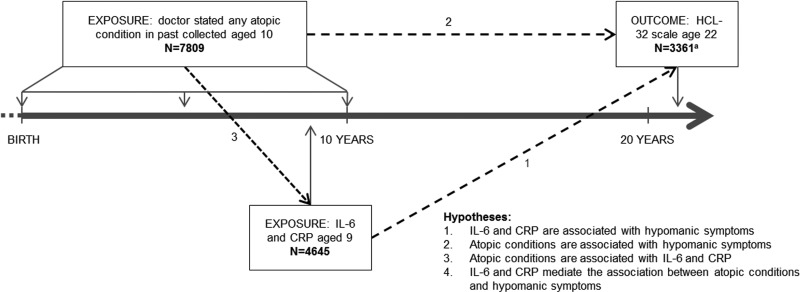
Timing of data collection, hypotheses and sample size. ^a^ Total participants who completed both interleukin-6 (IL-6) blood test and Hypomania Checklist (HCL-32), *n* = 1717. Total participants who completed both C-reactive protein (CRP) blood test and HCL-32, *n* = 1721. Total participants who completed atopic condition question and HCL-32, *n* = 2880.

Using data from ALSPAC we assessed associations between serum CRP and IL-6, aged 9 years, atopic conditions (asthma and eczema) before age 10 years, and lifetime experience of hypomanic symptoms assessed at 22 years. We hypothesized that atopic disorders and raised inflammatory markers would be associated with increased risk of hypomanic symptoms, and that inflammatory markers would mediate the association between atopic conditions and features of hypomania (Fig. 1).

## Method

### Description of cohort

The ALSPAC birth cohort is comprised of all live births in the County of Avon, UK, with expected due dates between April 1991 and December 1992. The initial cohort consisted of 14 062 live births, with 13 998 alive at 1 year (http://www.bristol.ac.uk/alspac/). From birth, parents completed regular questionnaires about all aspects of their child's health and development. From age 7 years, children attended a clinic to participate in various tests and interviews on an annual basis. ALSPAC has been used for a vast array of research topics (Fraser *et al.* 2013). Ethical approval for the study was obtained from the ALSPAC Ethics and Law Committee and Local Research Ethics Committees.

This study is based on the cohort of 4645 children who had analysable blood samples taken aged 9 years after excluding those with an infection around the time of blood collection, 7809 who answered questions about atopic conditions aged 10 years and 3361 who completed the Hypomania Checklist (HCL-32) (Fig. 1).

### Assessment of hypomanic symptoms

Features of hypomania were identified using the HCL-32 which was sent to participants when they were aged 22 years. The HCL-32 is a self-completed questionnaire for a lifetime history of hypomanic symptoms (Angst *et al.* 2005). It asks respondents to consider a time when they were in a ‘high or hyper’ state and endorse a number of statements about their emotions, thoughts and behaviours at this time. Examples of these 32 statements are: ‘I think faster’; ‘I make more jokes or puns when I am talking’; and ‘I take more risks in my daily life’. None of the statements describe psychotic experiences. It also asks about the duration of such episodes and their impact on family, social and work life. The HCL-32 has been used as both a continuous and categorical measure of hypomanic symptoms (Court *et al.* 2014; Carvalho *et al.* 2015). Although initially developed as a screening instrument for use in people diagnosed with depressive disorders, it is also a sensitive screening tool in non-clinical settings (Meyer *et al.* 2007).

We defined lifetime history of hypomanic symptoms in line with previous approaches for studies of this nature, namely: a score of 14 or more out of the possible 32 questions on hypomanic features, plus at least one response of either ‘negative consequences’ or ‘negative plus positive consequences’, plus a report that these mood changes causes some kind of reaction in others, plus a duration of ‘2–3 days’ or more. Overall, this definition of hypomanic symptoms, which includes severity, impairment and duration criteria, is more conservative than several other studies, which have tended to use only the threshold score of 14 (Hardoy *et al.* 2005; Meyer *et al.* 2007, 2014). We chose a duration criterion of 2–3 days or more because the 4-day threshold within the Diagnostic and Statistical Manual of Mental Disorders (DSM) excludes many individuals with bipolar disorder type II (Goldberg *et al.* 2009; Bauer *et al.* 2011; Parker *et al.* 2014), and because 2 days is known to be the modal duration of hypomania in bipolar II disorder (Benazzi, 2001; Angst & Cassano, 2005). Based on previous work in non-clinical samples, we expected 5–10% of respondents to satisfy our criteria for features of hypomania (Meyer *et al.* 2007; Holtmann *et al.* 2009). Hypomanic symptoms were not clinically verified in the cohort.

### Assays for inflammatory markers

The acute-phase protein, CRP; and the pleiotropic cytokine, IL-6, were assayed from non-fasting blood samples collected in participants aged 9 years. Parents of 7236 children gave informed consent for venepuncture. The procedure was successfully completed such that IL-6 and CRP were measured in 5076 individuals. Of these, 431 children reported an infection in the 7 days preceding venepuncture and were excluded from the cohort, as we were interested in immune activity in healthy individuals.

Serum was isolated after blood collection and stored in 1 ml aliquots at −80°C. Analysis of these samples was carried out in 2008 following a median of 7.5 years of storage, with no freeze–thaw cycles during storage. The automated particle-enhanced Tina-quant immunoturbidimetric assay (Roche UK) was used to measure high-sensitivity CRP. An enzyme-linked immunosorbant assay (ELISA) (R&D systems, UK) was used to measure IL-6. These methods have been reported elsewhere (Khandaker *et al.* 2014*a*). All inter- and intra-assay coefficients of variation for IL-6 and CRP were less than 5%. No other inflammatory markers were measured. The minimum detection limit for IL-6 was 0.1 pg/ml. This represents the lowest measureable analytic level that can be distinguished from zero. Those below this limit were assigned a value of zero (0.4% of the sample), and were also included in our analysis. In the total sample IL-6 values ranged from 0.1 to 20.1 pg/ml. The minimum detection limit for CRP was 0.03 mg/l. Of the participants, 29 (0.6% of the sample) were below this limit, and were assigned values of 0.01 pg/ml (*n* = 16) and 0.02 mg/l (*n* = 13); they were also included in the analysis. In the total sample, CRP values ranged from 0.01 to 45.17 mg/l (32 subjects over 10 mg/l).

### Assessment of atopic disorders

When participants were aged 10 years, parents completed a postal questionnaire which included the question: ‘Has a doctor ever actually said that your study child has asthma or eczema?’ Possible responses to this question which are used in this study were: no asthma or eczema (reference group); asthma only; eczema only; both asthma and eczema.

### Assessment of confounders

A number of potential childhood and parental confounders were defined *a priori*: age at the time of hypomania assessment; sex; ethnicity; socio-economic status (SES) (defined as paternal social class using the six categories of the UK Office of National Statistics classification system); psychological and behavioural problems at baseline [using the Strengths and Difficulties Questionnaire (SDQ), completed by parents when children were aged 7 years]; baseline body mass index (BMI) (measured at 9 years old), and mother's postnatal depression score [using the Edinburgh Postnatal Depression Scale (EPDS) recorded at 8 weeks post-partum]. These confounders were selected because they represent a range of measures of childhood adversity, all of which have been found to be associated with inflammatory processes in childhood and risk of adult mental disorders. We also ran an additional model adjusting for International Classification of Diseases (ICD)-10 diagnosis of depression at the age of 18 years made via the Clinical Interview Schedule-Revised (CIS-R) (Lewis *et al.* 1992). This was to check that any association was not purely driven by the previously reported relationship between inflammatory markers and depression symptoms in this cohort (Khandaker *et al.* 2014*a*).

### Statistical analysis

#### Inflammatory markers and hypomania

We used logistic regression to calculate the odds ratio (OR) and 95% confidence interval (CI) for hypomanic symptoms in individuals in the middle and top thirds of IL-6 and CRP compared with the bottom third (reference group). Linear trend was tested when tertiles of values of each inflammatory marker were treated as a linear term. Regression models were adjusted for age, sex, ethnicity, SES, SDQ score, BMI and EPDS score. Additionally, we calculated ORs for hypomanic symptoms for each standard deviation (s.d.) increase in IL-6 and CRP in childhood using these inflammatory markers as continuous variables (*z*-transformed values). A quadratic term (for example IL-6^2^) was included in these regression models to assess potential non-linearity.

#### Atopic disorders and hypomania

Multinomial logistic regression was used to calculate the OR and 95% CI for features of hypomania in those with asthma only, eczema only, and both asthma and eczema, compared with no asthma or eczema (reference group). Regression models were adjusted for age at time of HCL-32 completion, sex, ethnicity, SES, SDQ score, BMI and EPDS score.

#### Inflammatory markers and atopic disorders

Normality of distributions of IL-6 and CRP was achieved following natural logarithmic transformation. Linear regression was carried out using transformed values of inflammatory markers as the dependent variable comparing the groups with asthma, eczema, and both with participants with no atopic condition. Sex, ethnicity, SES, SDQ score, BMI and EPDS score were included as potential confounders.

#### Imputation of missing data

Following a complete-case analysis, we imputed missing data for all individuals who had completed the atopic disorder questions at the age of 9 years (*n* = 7809). We used sex, ethnicity, SES, SDQ score, BMI, EPDS score, IL-6 and CRP counts to predict missing HCL-32 score at the age of 22 years. We also included scores from the Psychosis-Like Symptom Interview and the CIS-R to improve the imputation model. These measures were completed when participants were aged 18 years. Multiple imputation using chained equations was completed using the *mi* and associated commands in Stata 13 (USA). A total of 100 imputed datasets were added. All regression models described above were repeated following multiple imputation, as well as models with additional adjustment for ICD diagnosis of depression at the age of 18 years.

## Results

### Baseline characteristics

Data on inflammatory biomarkers were available for 4645 participants (Table 1). Higher serum IL-6 levels at age 9 years were associated with female sex, non-white British ethnicity, lower SES, higher past psychological and behavioural problems and higher BMI. Atopic disorder diagnosis was known for 7809 children (Table 2). Children with atopic disorders had higher serum CRP and IL-6 levels at age 9 years. Children with asthma or both asthma and eczema were more likely to be boys, and they had higher maternal EPDS scores, childhood SDQ scores and BMI. Of those who completed the CIS-R at 18 years and HCL-32 at 22 years (*n* = 2298), 21 (1%) fulfilled criteria for hypomanic symptoms and ICD-10 depression.

**Table 1.** Baseline characteristics of the sample by tertiles of interleukin-6 at aged 9 years

	Bottom	Middle	Top	*p*^a^
Total *n* (%)	1548 (33.33)	1548 (33.33)	1548 (33.33)	
Mean age at outcome, years (s.d.)	21.96 (0.13)	21.95 (0.16)	21.96 (0.15)	0.79
Male, *n* (%)	923 (59.63)	791 (51.20)	649 (41.95)	<0.001
British white ethnicity, *n* (%)	1417 (91.54)	1393 (89.99)	1362 (87.98)	0.005
Father's occupation, non-manual, *n* (%)	783 (61.27)	703 (57.62)	670 (55.83)	0.02
Mean mother's EPDS score (s.d.)	5.69 (4.47)	6.00 (4.71)	5.93 (4.63)	0.17
Mean SDQ total score aged 7 years (s.d.)	6.29 (4.59)	6.46 (4.69)	6.88 (5.03)	0.005
Mean BMI at aged 9 years, kg/m^2^ (s.d.)	16.73 (1.89)	17.51 (2.51)	18.52 (3.40)	<0.001

s.d., Standard deviation; EPDS, Edinburgh Postnatal Depression Scale; SDQ, Strengths and Difficulties Questionnaire; BMI, body mass index.

a *p* Values from χ^2^ test for difference in proportions, and analysis of variance for difference in means.

**Table 2.** Baseline characteristics of the sample by atopic disorder status

	Asthma	Eczema	Both	No atopy	*p*^a^
Total *n* (%)	1124 (14.39)	994 (12.73)	574 (7.35)	5117 (65.53)	
Mean age at outcome, years (s.d.)	21.95 (0.13)	21.95 (0.15)	21.97 (0.16)	21.96 (0.15)	0.38
Male, *n* (%)	661 (58.81)	439 (44.16)	311 (54.18)	2530 (49.44)	<0.001
British white ethnicity, *n* (%)	1014 (90.21)	907 (91.25)	520 (90.59)	4666 (91.19)	0.74
Father's occupation, non-manual, no. (%)	472 (53.33)	474 (58.81)	272 (58.24)	2414 (58.25)	0.049
Mean mother's EPDS score (s.d.)	6.38 (4.99)	5.74 (4.55)	6.20 (4.68)	5.68 (4.55)	<0.001
Mean SDQ total score aged 7 years (s.d.)	7.19 (4.93)	6.76 (4.85)	7.55 (5.61)	6.64 (4.85)	<0.001
Mean BMI at aged 9 years, kg/m^2^ (s.d.)	17.94 (3.05)	17.81 (2.79)	17.82 (3.05)	17.56 (2.83)	<0.001
Median CRP, mg/l (IQR)	0.20 (0.11–0.51)	0.23 (0.13–0.54)	0.22 (0.11–0.69)	0.20 (0.11–0.47)	0.019
Median IL-6, pg/ml (IQR)	0.82 (0.49–1.39)	0.83 (0.54–1.38)	0.84 (0.52–1.46)	0.74 (0.46–1.31)	0.0069

s.d., Standard deviation; EPDS, Edinburgh Postnatal Depression Scale; SDQ, Strengths and Difficulties Questionnaire; BMI, body mass index; CRP, C-reactive protein; IQR, interquartile range; IL-6, interleukin-6.

a *p* Values from χ^2^ test for difference in proportions, analysis of variance for difference in means and Kruskal–Wallis test for difference in medians.

### Inflammatory markers and the risk of hypomanic symptoms at age 22 years

Of participants who had a blood test at the age of 9 years, 1717 with IL-6 and 1721 with CRP results completed the HCL-32 at 22 years. In total, 116 (6.8%) fulfilled criteria for hypomanic symptoms via HCL-32. Those in the top third of IL-6 at 9 years had an increased risk of hypomanic symptoms aged 22 years, compared with those in the bottom third. This relationship became stronger after adjusting for age, sex, ethnicity, SES, SDQ, BMI and EPDS; adjusted OR for hypomanic symptoms for participants in the top third of IL-6, compared with the bottom third, was 1.87 (95% CI 1.07–3.27, *p* = 0.028). There was also evidence of a relationship between IL-6 and hypomanic symptoms when IL-6 values divided into thirds were treated as a linear term (OR for trend 1.36, 95% CI 1.03–1.78, *p* = 0.027). Using IL-6 as a continuous measure, the adjusted OR for hypomanic symptoms was 1.21 (95% CI 1.03–1.43, *p* = 0.021) for each 1 s.d. increase in IL-6. There was no evidence to support a non-linear relationship. There was no evidence of an association between CRP levels and hypomanic symptoms. Evidence for an association between IL-6 and hypomanic symptoms remained unchanged after multiple imputation for missing data (Table 3). In the multiple imputation dataset the association between IL-6 and hypomanic symptoms remained after additional adjustment for depression (OR for top third compared with bottom third 1.70, 95% CI 1.03–2.81, *p* = 0.038).

**Table 3.** Serum IL-6 and CRP tertiles at age 9 years and the odds of hypomania aged 22 years

				Hypomania symptoms: OR (95% CI) [*p*]			
	Tertile	*n*	Hypomania, *n* (%)	Unadjusted	Model 1^a^	Model 2^b^	Model 3^c^
*n*^d^				1717	1699	1201	7809
IL-6							
	Bottom	573	30 (5.24)	1	1	1	1
	Middle	551	39 (7.08)	1.38 (0.84–2.25) [0.202]	1.51 (0.91–2.48) [0.104]	1.59 (0.91–2.79) [0.106]	1.53 (0.98–2.42) [0.064]
	Top	593	47 (7.93)	1.55 (0.97–2.50) [0.069]	1.78 (1.09–2.89) [0.020]	1.87 (1.07–3.27) [0.028]	1.77 (1.10–2.85) [<0.001]
	Linear trend	1717	116 (6.76)	1.24 (0.98–1.56) [0.069]	1.32 (1.04–1.67) [0.021]	1.36 (1.03–1.78) [0.027]	1.31 (1.04–1.65) [0.022]
CRP							
	Bottom	622	52 (8.36)	1	1	1	1
	Middle	532	34 (6.39)	0.75 (0.48–1.17) [0.207]	0.83 (0.53–1.31) [0.428]	0.87 (0.52–1.48) [0.614]	0.84 (0.52–1.34) [0.480]
	Top	567	30 (5.29)	0.61 (0.38–0.97) [0.038]	0.69 (0.43–1.11) [0.125]	0.71 (0.40–1.28) [0.232]	1.04 (0.67–1.62) [0.872]
	Linear trend	1721	116 (6.74)	0.78 (0.62–0.98) [0.033]	0.83 (0.65–1.05) [0.128]	0.85 (0.64–1.13) [0.266]	1.02 (0.81–1.29) [0.877]

IL-6, Interleukin-6; CRP, C-reactive protein; OR, odds ratio; CI, confidence interval; HCL-32, Hypomania Checklist; BMI, body mass index.

a Complete case analysis adjusted for age at time of HCL-32 completion, sex and ethnicity.

b Complete case analysis adjusted for age at time of HCL-32 completion, sex, ethnicity, socio-economic status, psychological and behavioural problems, BMI and maternal postnatal depression.

c Values imputed for all participants who answered the question about atopy aged 10 years (*n* = 7809) and adjusted for age at time of HCL-32 completion, sex, ethnicity, socio-economic status, psychological and behavioural problems, BMI and maternal postnatal depression.

d Number of participants in each model.

### Atopic disorders and the risk of hypomanic symptoms at age 22 years

At age 22 years, 207 of 2880 respondents with atopic condition and HCL-32 data met our pre-defined criteria for lifetime hypomanic symptoms (7.2%). There was no evidence for an increased odds of hypomanic symptoms in children with asthma, eczema or both asthma and eczema, compared with those with no atopic illness. Adjustment for potential confounders had minimal influence on the magnitude of these ORs (Table 4). Multiple imputation of missing values did not alter these results.

**Table 4.** Atopic disorders and the odds of hypomania aged 22 years

			Hypomania symptoms, OR (95% CI) [*p*]			
	*n*	Hypomania, no. (%)	Unadjusted	Model 1^a^	Model 2^b^	Model 3^c^
*n*^d^			2880	2857	1872	7809
None	1929	134 (6.95)	1	1	1	1
Asthma	348	27 (7.76)	1.13 (0.73–1.73) [0.591]	1.07 (0.69–1.66) [0.775]	1.27 (0.77–2.11) [0.359]	1.03 (0.66–1.60) [0.903]
Eczema	385	28 (7.27)	1.05 (0.69–1.60) [0.831]	1.07 (0.71–1.65) [0.766]	1.22 (0.74–2.01) [0.444]	1.06 (0.69–1.62) [0.801]
Both	218	18 (8.26)	1.21 (0.72–2.01) [0.476]	1.17 (0.70–1.96) [0.561]	1.00 (0.52–1.93) [0.999]	1.09 (0.65–1.83) [0.757]

OR, Odds ratio; CI, confidence interval; HCL-32, Hypomania Checklist; BMI, body mass index.

a Complete case analysis adjusted for age at time of HCL-32 completion, sex and ethnicity.

b Complete case analysis adjusted for age at time of HCL-32 completion, sex, ethnicity, socio-economic status, psychological and behavioural problems, BMI and maternal postnatal depression.

c Values imputed for all participants who answered the question about atopy aged 10 years (*n* = 7809) and adjusted for age at time of HCL-32 completion, sex, ethnicity, socio-economic status, psychological and behavioural problems, BMI and maternal postnatal depression.

d Number of participants in each model.

### Atopic disorders and inflammatory markers in childhood

Data on atopic disorders and IL-6 were available for 3891 individuals. Diagnoses of asthma (*β* = 0.081, s.e. = 0.041, *p* = 0.048), eczema (*β* = 0.102, s.e. = 0.042, *p* = 0.014) or both (*β* = 0.127, s.e. = 0.055, *p* = 0.020) before the age of 10 years were all associated with increased serum IL-6 at the age of 9 years. However, following adjustment for sex, ethnicity, SES, EPDS, SDQ and BMI there was no evidence of an association. In total, 3898 individuals had both CRP and atopic disorder information. Again, each atopic illness was associated with elevated CRP: asthma (*β* = 0.121, s.e. = 0.054, *p* = 0.026); eczema (*β* = 0.155, s.e. = 0.056, *p* = 0.006) and both (*β* = 0.167, s.e. = 0.073, *p* = 0.022), but there was no evidence of association following adjustment.

## Discussion

To our knowledge, this is the first study to demonstrate a longitudinal dose–response relationship between childhood IL-6 levels at the age of 9 years and features of hypomania in early adulthood. The relationship persists after adjustment for potential confounders such as age, sex, ethnicity, SES, and psychological and behavioural problems preceding the measurement of IL-6, BMI and maternal postnatal depression. The association was also independent of diagnosed depression at 18 years. We did not find an association between CRP and hypomanic symptoms, as defined by HCL-32. We also found no evidence of association between asthma or eczema before the age of 10 years and features of hypomania. There was no evidence of an association between atopic disorders and IL-6 or CRP following adjustment for sex, ethnicity, SES, BMI, SDQ and EPDS scores.

The finding of an association between IL-6 and features of hypomania echoes the results from a previous study from the ALSPAC cohort that reported an association between childhood IL-6 and depressive and psychotic symptoms at the age of 18 years (Khandaker *et al.* 2014*a*). Combined, this body of work suggests that aberrant immune response in childhood might influence the risk of a range of major mental disorders. It is recognized that hypomanic symptoms in early adulthood are associated generally with an increased risk of Axis I disorders, and specifically with developing bipolar disorder (Zimmermann *et al.* 2009; Päären *et al.* 2014). The result is particularly interesting as it suggests an association of subtle changes in cytokine levels in early life with hypomanic symptoms assessed in adulthood, where previous research has found immune activation returns to normal between affective episodes in bipolar disorder (Kunz *et al.* 2011; Stertz *et al.* 2013).

The lack of evidence for an association between atopic illness and hypomanic symptoms is surprising given a number of cross-sectional studies (Goodwin *et al.* 2003; Beyer *et al.* 2005; Jerrell *et al.* 2010) and longitudinal studies (Liang & Chikritzhs, 2013; Chen *et al.* 2014; Lin *et al.* 2014) have reported an association between asthma and bipolar disorder. This may be because previous studies have examined the severe end of the bipolar spectrum (hospitalization for bipolar disorder) and this association may not hold when our broad definition of hypomania is used. The only study to examine asthma in childhood and later-onset bipolar disorder was a cohort with a new diagnosis of asthma between the ages of 10 and 15 years, which found that individuals with asthma had more than a twofold risk of bipolar disorder (Chen *et al.* 2014). It is recognized that individuals who have persistent asthma through adolescence, or develop asthma in adolescence, have worse physical health outcomes than children whose asthma resolves in early adolescence (Sears *et al.* 2003). It is possible that this is also true in terms of psychiatric outcomes, and that with a cut-off for asthma and eczema before the age of 10 years we have examined a highly heterogeneous group in terms of severity and underlying immunopathology (Wenzel, 2006).

There is limited current understanding of mechanisms by which changes in inflammatory pathways could induce hypomanic symptoms. However, it is notable that serotonin- and tryptophan-degrading enzymes are activated by inflammatory cytokines such as IL-6 (Müller & Schwarz, 2007). As well as influencing monoamine cascades, cytokine production has effects on cholinergic, muscarinic and gutamatergic systems which are associated with a range of affective and psychotic symptoms (Berk *et al.* 2011; Khandaker & Dantzer, 2016). The potential effects of IL-6 in particular are complex; it is implicated in activation of the immune system (as a proinflammatory cytokine), but it is also involved in the anti-inflammatory regulation of neural, regenerative and metabolic activity (Scheller *et al.* 2011). Peripheral cytokines such as IL-6 can communicate with the brain in a number of ways (Khandaker & Dantzer, 2016). Immunoactivation in mice and healthy volunteers by injection of lipopolysaccharide has been reported to increase circulating proinflammatory cytokine levels (IL-1*β*, IL-6) as well as producing symptoms of anxiety and reduced cognitive performance (Reichenberg *et al.* 2001; Rossi *et al.* 2012). A study of human volunteers suggests inflammation causes mood changes through alterations in subgenual cingulate activity and mesolimbic connectivity, which is in part mediated by peripheral IL-6 (Harrison *et al.* 2009). An immunological understanding of major mental illness could potentially lead to novel approaches to diagnosis, prevention and treatment.

### Potential limitations

Over 60% of those with IL-6 data did not complete the HCL-32 aged 22 years. However, the association between IL-6 and hypomanic symptoms remained unchanged after multiple imputation for missing data which increases confidence in the observed results. There was no evidence that median values of IL-6 for those who completed HCL-32 were different from non-completers. Of those participants who answered the atopic disorders question, individuals with a completed HCL-32 aged 22 years were more likely to be female, have lower SDQ scores aged 7 years, have mothers with lower EPDS scores and fathers in non-manual occupations. It is likely that attrition resulted in an underestimation of prevalence of hypomanic symptoms, as these factors were associated with a lower risk of reporting features of hypomania.

The outcome measure, the HCL-32, may be subject to reporting bias because it is a self-report measure that asks sensitive questions in areas such as risk taking, sexual activity and alcohol use. However, this instrument is well validated in clinical young adult population groups as a screening tool for bipolar disorder (Meyer *et al.* 2007). There have not yet been sensitivity and specificity tests of the HCL-32 as a categorical measure which includes duration and impact on functioning. However, it is likely that by including questions on these features we have improved sensitivity for a diagnosis of hypomania compared with methods which have previously relied solely on a recommended (arbitrary) cut-off of endorsing 14 from the 32 mood questions (Carvalho *et al.* 2015). It is also unlikely that false positives would occur differentially for different tertiles of IL-6 or CRP, or by asthma/eczema diagnosis. Prevalence estimates of hypomania in non-clinical samples using this format of HCL-32 also appear to be comparable with bipolar disorder prevalence in other sources (Meyer *et al.* 2007). Clinical verification of hypomania and information on bipolar disorder diagnoses would strengthen our conclusions, but it is likely that a number of individuals with hypomania symptoms will progress to a diagnosis and these symptoms are associated with a range of adverse psychiatric outcomes (Zimmermann *et al.* 2009; Päären *et al.* 2014). The items in the HCL-32 do not relate to symptoms of psychosis or depression, and after additional adjustment for depression the association remained, so the relationship cannot be driven by this previously identified association (Khandaker *et al.* 2014*a*).

Having a diagnosis of asthma or eczema was defined by parental report. The study may have been improved by a test confirming this diagnosis. However, the question ‘has a doctor ever actually said that your study child has asthma or eczema?’ has been shown to be highly sensitive and specific when compared with linked general practitioner records (Cornish *et al.* 2014) and there is unlikely to be any differential misclassification of this exposure. Prevalence of atopic conditions in the ALSPAC cohort by aged 10 years is similar to other UK estimates (Asher *et al.* 2006; Shamssain, 2007).

It is possible that some of the differences in IL-6 and CRP levels detected are due to diurnal variation in non-fasting blood samples, which would increase chances of null findings (Scheiermann *et al.* 2013). However, this potential measurement error is likely to be random in relation to the hypomanic symptoms outcome. Measurement error might also account for failure to detect an association between CRP and hypomanic symptoms, because IL-6 stimulates the acute-phase expression of CRP (Castell *et al*. 1989). It is also possible that we failed to exclude all of those children who were acutely unwell on the day of the blood test, or had elevated inflammatory markers due to residual illness. This is unlikely, as the majority of CRP and IL-6 results were within normal range. We acknowledge that immunoglobulin E or IL-4 may be better markers for type 1 allergic hypersensitivity, but we used IL-6 and CRP as general markers of systemic inflammation.

## Conclusions

The results of this study further support the hypothesis that inflammatory abnormalities are associated with a range of mental disorders, and that these abnormalities are present from childhood. It appears that there are subtle, but longstanding abnormalities in IL-6 detectable long before the development of hypomanic symptoms.
